# Seroprevalence of SARS-CoV-2 immunoglobulin G antibody during COVID-19 pandemic in Fayoum District, Egypt: a community-based pilot survey

**DOI:** 10.11604/pamj.2023.45.22.36513

**Published:** 2023-05-05

**Authors:** Salwa Bakr, Eman Mahmoud Ezzat, Karem Mohamed Salem, Mohamed Masoud, Hossam Eldin Mahmoud Abdelaziz

**Affiliations:** 1 Department of Clinical Pathology/Hematology and Transfusion Medicine, Faculty of Medicine, Fayoum University, Fayoum, Egypt,; 2 Department of Internal Medicine, Faculty of Medicine, Fayoum University, Fayoum, Egypt,; 3 Department of Public Health and Community Medicine, Faculty of Medicine, Fayoum University, Fayoum, Egypt

**Keywords:** Coronavirus, SARS-CoV-2, COVID-19, seroconversion, Egypt

## Abstract

**Introduction:**

controlling the worldwide pandemic, coronavirus disease (COVID-19), could be impossible due to the hesitancy about the available vaccines and the difficulty to implement strict restrictions. Little information is available about herd immunity in the highly vulnerable region of North East Africa, Egypt. The objective of this study was to assess the seroprevalence of SARS-CoV-2 during the pandemic in one of the highly vulnerable populations in Egypt, the Fayoum district of Fayoum Governorate. Additionally, to assess the predictive value of symptoms and other associated risk factors towards a positive COVID-19 test.

**Methods:**

in this cross-sectional community-based pilot study, immunoglobulin G (IgG) antibodies that are specific for the SARS-CoV-2 spike (S1-RBD) protein were tested during the period from February 2021 to July 2021.

**Results:**

out of 155 participants, 60.6% were SARS-CoV-2 seropositive. Out of symptomatic and asymptomatic individuals, 76.5% and 56.2% were seropositive, respectively. Surprisingly, only one individual had received the COVID-19 vaccine. Previous history of COVID-19; such as symptoms and gender are statistically significant predictors of high seroconversion independent of age, comorbidities, and level of education.

**Conclusion:**

this study which disclosed unexpectedly high SARS-CoV-2 seroconversion among the Egyptians, might provide a clear insight into COVID-19 transmission patterns and state of immunity. Further study with a larger sample size on a large scale is required to represent the whole local population.

## Introduction

The coronavirus disease outbreak in late 2019 (COVID-19) has emerged as a worldwide pandemic caused by severe acute respiratory syndrome coronavirus 2 (SARS-CoV-2) [1]. The virus outbreak began in December 2019 in Wuhan, China, and spread largely through droplets and surface contact routes [2]. In mid-March 2020, the World Health Organization (WHO) categorized this disease as a pandemic level [3]. Although the severity of its acute respiratory illness varied between individuals from mild symptoms of fever, and cough, with or without shortness of breath to fatal respiratory distress, the infection can also be asymptomatic [4]. The disease pathogenesis develops through the interaction between the outer envelope of the virion particle (spike protein or S-protein) and the angiotensin-converting enzyme 2 (ACE2) in the respiratory cells of the infected individuals [5].

Although the correlation between immune response and COVID-19 protection is not fully clear [6], many studies affirmed that SARS-CoV-2 seropositive individuals are less likely to experience a subsequent infection or clinical manifestation of the disease than seronegative ones [7-9]. In numerous studies, nearly 80-90% reduction in infection was indicated among individuals with antibodies compared with those without antibodies [6]. Although IgG antibodies may not be detected in approximately 5-10% of individuals post-infection [10,11], the response of humoral immunity seems to remain integral even with a loss of specific antibodies over time owing to the persistence of memory B-cells [12].

In 2021, the Centers for Disease Control and Prevention (CDC) declared that testing for anti-SARS-CoV-2 antibodies could be a useful public health tool for a better understanding of the virus epidemiology, analysis of transmission patterns, and implementation of vaccination programs [6]. Serologic tests for antibodies with preferential use of semi-quantitative tests can identify individuals with prior COVID-19 infection with negative real-time reverse transcription polymerase chain reaction (rRT-PCR) test results and help in the early identification of asymptomatic subclinical infections thereby to convey the population´s herd immunity [13,14], providing that the serology tests for detecting antibodies against specific SARS-CoV-2 proteins are adequately validated [15].

In Egypt, a country in East North Africa, the first lab-confirmed case of SARS-CoV-2 was recorded in mid-February 2020. Then after, control measures; such as isolation of the reported cases and tracing of the close contacts were implemented. Consequently, the major public health and social measures to control the outbreak; such as the closure of schools and universities, partial curfew, and international air flight bans were applied by mid-March 2020. Since 30^th^ May 2020, wearing face masks became mandatory. However, air traffic had been opened. Later on, public areas; such as places of worship and restaurants were reopened, and the curfew was lifted on 27^th^ June 2020 [16]. Vaccination for healthcare workers and high-risk groups was initiated in March 2021 [17]. The threshold for herd immunity or vaccination proportion necessary for disease elimination depends on the basic reproductive number (R0) of the infection, this threshold could be estimated as 1-1/R0 [18]. In Egypt, according to the published estimation of R0 (above 2), this threshold is expected to be above 50% [16,19]. By 12 November 2021, a total of 34,871,428 vaccine doses have been administered. As of 19 November 2021, 346,808 confirmed cases of COVID-19 with 19,707 deaths were reported to the WHO [20]. As of 25^th^ October 2021, CDC enlisted Egypt among the highly pandemic regions in the world (level 4) [21]. Although the Egyptian Ministry of Health targets COVID-19 vaccination of 40% of its population by the end of 2021, vaccine hesitancy is one of the pandemic problems in many countries including Egypt owing to false claims and misinformation about the available vaccines [22].

In literature, little information is available about herd immunity among the highly vulnerable population of North East Africa, Egyptian. Hence, the present study aimed to assess the seroprevalence of SARS-CoV-2 during the pandemic at one of the highly vulnerable destinations in Egypt, the Fayoum district of Fayoum Governorate. Additionally, the study aimed to assess the predictive value of symptoms and other associated risk factors toward a positive COVID-19 test.

## Methods

**Study design:** the present cross-sectional, community-based pilot survey study was conducted in the Fayoum district of Fayoum Governorate during the period from February 2021 to July 2021 after taking the approval of the local ethical committee at the Faculty of Medicine, Fayoum University (R250). Written informed consent was obtained from all study participants. All study procedures were following the ethical standards of the institutional and/or national research committee and with the 1964 Declaration of Helsinki.

**Sample size calculation:** Fayoum governorate which is located in Upper North Egypt has a total population of approximately 3,897,601 as estimated on 1/1/2021 with about 2,992,459 living in rural areas and 905,142 living in urban areas [23]. The sample size of the main study was calculated using Epi Info 7. A sample size of (323) was calculated, assuming 30% as the prevalence of antibodies against SARS-CoV-2 where a confidence interval of 95% and precision of 5%. To achieve the same precision, the method of stratified and cluster sampling was considered and the calculated sample size was tripled to reach (969). To overcome the problems of missing data and non-responses, the sample size was increased by 10% reaching up to (1066). According to Connelly *et al*. 2008 we indented to include at least 10% of the calculated sample size in our pilot study [24]. The final study sample was 155 participants.

**Sampling procedures:** Fayoum governorate includes six administrative districts (Fayoum, Sinnuris, Tamiya, Etsa, Abshoay, and Youssef Sadiek). Within the randomly selected Fayoum district, a village was randomly chosen that was recorded in census data. Within the selected village a street was chosen at random with the decision to proceed in one direction. Then, each first and fourth house on the street was randomly chosen.

**Data collection:** the study population was interviewed using a structured Arabic questionnaire consisting of two sections. The first one requires sociodemographic data. The second section includes the medical history regarding previous COVID-19 infection, vaccination, and history of comorbidities.

**Laboratory testing:** blood sample (2 mL into a plain tube) was collected from all participants after taking written informed consent. Stand serum samples were left at room temperature until the separation of the serum by centrifugation at 4°C, at a speed of 3000 rev/min. keeping the separated sera were stored at 20°C until its analyses time, which was then thawed for testing SARS-CoV-2 antibodies using DIALAB Enzyme-Linked Immunosorbent Assay (ELISA) for qualitative/semi-qualitative detection of IgG antibodies specific for the SARS-CoV-2 Spike S1-RBD protein (DIALAB GmbH, Austria), as per the manufacturer´s instruction.

**Statistical analysis:** statistical data analysis was performed using software statistical computer package version 22 of Statistical Package of Social Science (SPSS Inc, Chicago, IL, USA). Descriptive statistics were performed for illustrating the basic characteristics of the study participants and the estimate of seroprevalence. For describing quantitative data, mean and standard deviation (SD) were calculated. While qualitative data were presented in the form of numbers and percentages. Seroprevalence to SARS-CoV-2 was estimated as a proportion of participants with seropositive results.

Inferential statistics were performed to assess the association between risk factors and symptoms with seroprevalence. An independent t-test was used in comparing seropositive and seronegative groups as regards age. Chi-square (χ^2^) was used as a test of significance regarding categorical data. Crude and adjusted odds ratios and their 95% confidence intervals (CI) to estimate the risk of seroconversion for different predictors were calculated using univariate and multivariate logistic regression, respectively. For interpretation of results of tests of significance, significance was adopted at P < 0.05.

**Ethical approval and consent to participate:** the study was approved by the ethical committee of the Faculty of Medicine, Fayoum University. Written informed consent was obtained. All study procedures were following the ethical standards of the institutional and/or national research committee and with the 1964 Declaration of Helsinki.

## Results

This cross-sectional study included 155 participants during the COVID-19 pandemic. Most of the study participants, 66.5% (103/155) were females. Their age ranged from 9 to 84 years with a mean ± SD of 35.9 ± 15.4. Of most of the participants, 70.3% (109/155) could read and write. Housewives represented 60% of our sample population. The majority of the study participants, 90.3% (140/155) had no history of contact with COVID-19 patients. Less than one-quarter of the participants, 21.9% (34/155) experienced previous COVID-19-like symptoms. Out of them, seven cases had COVID-19-like symptoms during the 3 weeks before the survey. SARS-CoV-2 infection was confirmed in 11 cases. Only one subject had a full dose of COVID-19 vaccination (Sinopharm). About one-third, 33.5% (52/155) had at least one chronic disease (Table 1).

**Table 1:** characteristics of study participants

Variables		N ( N = 155)	Percentage (%)
Gender	Male	52	33.5%
	Female	103	66.5%
Occupation	Housewives	93	60.0%
	Manual workers	11	7.1%
	Clerks	6	3.9%
	Farmers	17	11.0%
	Students	28	18.1%
Level of education	Currently educated	28	18.1%
	Read and write	109	70.3%
	Secondary	12	7.7%
	University	6	3.9%
Contact a patient with COVID-19	Yes	15	9.7%
	No	140	90.3%
Previous COVID-19 symptoms	Yes	34	21.9%
	No	121	78.1%
Confirmed COVID-19	Yes	11	7.1%
	No	144	92.9%
Chronic comorbidities	Yes	52	33.5%
	No	103	66.5%
COVID-19 vaccination	Yes	1	0.6%
	No	154	99.4%

Our results revealed that the majority of our sample population, 60.6% (94/155) developed SARS-CoV-2 antibodies (IgG) seroconversion (76.5% of symptomatic participants, and 56.2% of asymptomatic ones were seropositive, p=value 0.036). This seroconversion was statistically significantly associated with gender and type of occupation. As IgG seroprevalence was higher in females than males (68% and 46.2% respectively), and among clerks and housewives than manual workers (83.3%, 66.7%, and 27.3% respectively). On the other hand, neither age nor level of education had any statistically significant difference between seropositive and seronegative participants (Table 2). Though the seroprevalence was higher among the participants who reported the potential exposure to contact a patient with SARS-CoV-2 infection compared to those unexposed (80% vs. 58.6%), and among individuals with confirmed COVID-19 disease than those without confirmation (81.8% vs. 59%), there were no statistically significant differences (p=0.119 and p=0.154 respectively) (Table 2).

**Table 2:** relation between seroprevalence and characteristics of study participants

		Serology				Odds ratio (95% CI)	P-value
		Positive (N=94)		Negative (N=61)			
**Age (Mean ± SD)**		35.9 ±16.1		36.0 ±14.5		NA	0.964
Gender	Male	24	46.2%	28	53.8%	r	
	Female	70	68.0%	33	32.0%	2.475 (1.248-4.907)	0.009*
Occupation	Manual workers	3	27.3%	8	72.7%	r	
	Farmers	8	47.1%	9	52.9%	2.370 (0.463-12.138)	0.300
	Students	16	57.1%	12	42.9%	3.556 (0.755-16.313)	0.103
	Housewives	62	66.7%	31	33.3%	5.333 (1.322-21.524)	0.019*
	Clerk	5	83.3%	1	16.7%	13.332 (1.069-166.374)	0.044*
Level of education	Currently educated	16	57.1%	12	42.9%	1.333 (0.406-4.379)	0.635
	Read and write	69	63.3%	40	36.7%	1.725 (0.633-4.702)	0.287
	Secondary/university	9	50.0%	9	50.0%	r	
Contact a patient with COVID-19	No	82	58.6%	58	41.4%	r	
	Yes	12	80.0%	3	20.0%	2.829 (0.784-10.476)	0.119
Previous COVID-19 symptoms	No	68	56.2%	53	43.8%	r	
	Yes	26	76.5%	8	23.5%	2.533 (1.061-6.046)	0.036*
Confirmed COVID-19	No	85	59.0%	59	41.0%	r	
	Yes	9	81.8%	2	18.2%	3.124 (0.651-14.980)	0.154
Comorbidities	No	62	60.2%	41	39.8%	r	
	Yes	32	61.5%	20	38.5%	1.058 (0.534-2.097)	0.872

* Significant at p<0.05; CI: confidence interval; NA: not applicable; r: reference category (lowest risk category)

Our results revealed that individuals with a history of chronic diseases had a similar rate of SARS-CoV-2 seroconversion when compared to those without any comorbidities (61.5% vs. 60.2%) (Table 2). Multiple forward stepwise logistic regression analyses identified female gender (OR =2.583, 95% CI: 1.278-5.222, p=0.008), and individuals with previous COVID-19 symptoms (OR = 2.457, 95% CI: 1.005-6.009, p=0.049) to be statistically significant predictors for high SARS-CoV-2 seroconversion (Table 3). This study results pointed out that clinical manifestation of anosmia was highly predictive to SARS-CoV-2 seroprevalence (p=0.004) rather than other reported clinical manifestations for COVID-19 infection including fever, cough, dyspnea, bony ache or diarrhea (Table 4).

**Table 3:** multiple forward step-wise regression analysis

	P-value	Odds ratio	95% CI	
			Lower limit	Upper limit
Gender	0.008	2.583	1.278	5.222
Previous COVID-19 symptoms	0.049	2.457	1.005	6.009
Constant	0.211	0.688		

* Significant at p<0.05; CI: confidence interval

**Table 4:** the predictive value of symptoms toward a positive COVID-19 serology test

		Serology				Odds ratio (95% CI)	P-value
		Positive (N=94)		Negative (N=61)			
Fever	No	78	60.0%	52	40.0%	r	0.708
	Yes	16	64.0%	9	36.0%	1.185 (0.487-2.883)	
Cough	No	80	59.3%	55	40.7%	r	0.362
	Yes	14	70.0%	6	30.0%	1.604 (0.581-4.431)	
Dyspnea	No	86	59.3%	59	40.7%	r	0.212
	Yes	8	80.0%	2	20.0%	2.744 (0.563-13.383)	
Anosmia	No	83	57.6%	61	42.4%	NA	0.004*
	Yes	11	100.0%	0	0.0%		
Bony ache	No	79	58.1%	57	41.9%	r	0.091
	Yes	15	78.9%	4	21.1%	2.706 (0.853-8.582)	
Diarrhea	No	87	59.2%	60	40.8%	r	0.146
	Yes	7	87.5%	1	12.5%	4.828 (0.579-40.257)	

* Significant at p<0.05; NA: non-applicable; CI: confidence interval

## Discussion

Population-wide studies on SARS-CoV-2 seroprevalence in numerous systemic reviews and meta-analyses indicated worldwide variations in the seroconversion reflecting the dissimilarities in community transmission that might be based on geographic latitudes and/or climate, local resources, public health behavioral responses, and the underlying built environment [25,26]. In many countries, the execution of strict restrictions for controlling the COVID-19 pandemic seems difficult and could be impossible including Egypt [27]. Hence, the present study is designed to evaluate the seroprevalence of anti-SARS-CoV-2 antibodies among one of the worldwide claimed vulnerable populations of Egypt, where huge socioeconomic and cultural differences are present.

In a pooled analysis of systemic review and meta-analysis study aimed to estimate SARS-CoV-2 seroprevalence among residents of African countries during the pandemic from December 2020 to April 2021, the lowest seroprevalence (12%) was in studies conducted in East Africa compared to North, West, and Southern Africa (13%, 25%, and 34% respectively) [28]. This finding is contradictory to our results conducted among residents of North East Africa in Egypt during the nearby time point of the pandemic, as the majority of our sample population (60%) developed SARS-CoV-2 seroconversion independent of age, level of education, comorbidities, contact with infected individuals, or COVID-19 vaccination. Unlike similar other studies in Africa [28], the present study found that age, level of education [29], comorbidities [30], and vaccination intake did not influence the overall seroprevalence. Surprisingly, 90.3% had no history of contact with a patient with COVID-19.

Our results showed that previous COVID-19-like symptoms were one of the perfect predictors for high SARS-CoV-2 seroconversion among Egyptians. Similarly was reported in Buenos Aires City, Argentina where individuals with confirmed COVID-19 infection, increased two-fold the chance of being SARS-CoV-2 seropositive [13]. SARS-CoV-2 seropositive in our study was statistically significantly associated with both genders being higher among females, and type of occupation being higher among clerks than housewives. In previous studies in Africa, a numerically higher seroconversion was listed in females, whereas some other studies found high seroconversion among males. However, the insignificant differences in gender were documented in reports of the WHO, as well as, the African CDC (28). Our finding might be explained by the double number of randomly participating females compared to males.

Most (72.3%) of the SARS-CoV-2 seroconversion among this study´s participants were asymptomatic/subclinical infections. The lower incidence of COVID-19 prevalence in Egypt might be attributed to temperature and humidity, low amount of screening testing, the huge population, the compulsory Bacille Calmette-Guerin (BCG) vaccine use [31], the young age of populations, misdiagnosis [32], and host genetics [33].

Genetic variation has also been proven to alter the resistance of indigenous Africans to a variety of infectious diseases [34]. In support, this study could provide better insight into the immunity among one of such nations, Egyptian, against SARS-CoV-2 during the pandemic time. For future decision-making on epidemiological control measures, our study might help to estimate when (or if) we reach a state of herd immunity. Although the availability of a confirmed objective justification of seropositivity whether due to natural immunity or vaccination is a limitation of this study due to the lack of resources for performing any further confirmatory tests such as nucleocapsid protein-based immunoassay and molecular-based assay, as well as, our incapability to review vaccination certificates or PCR reports of the participants, detailed history taking with a conclusive reporting of only one vaccinated participant is likely to be valuable in predicting a clear justification.

## Conclusion

This pilot cross-sectional study which might provide a clear insight into COVID-19 transmission patterns and the state of herd immunity in one of the highly vulnerable populations of the Fayoum district in Egypt disclosed unexpectedly high SARS-CoV-2 seroconversion in the region. Further study with a larger sample size on a large scale is required to represent the whole local population.

### What is known about this topic

- COVID-19 transmission patterns and the state of immunity in the highly vulnerable population of North East Africa in Egypt are still ambiguous;
- Controlling the COVID-19 outbreak could be impossible in such regions due to the difficulty to implement strict restrictions and the hesitancy about the available vaccines due to misinformation and false claims.

### What this study adds

- Estimating SARS-CoV-2 seroconversion during the pandemic in one of the highly vulnerable destinations of Egypt is the only helpful approach to determine when (or if) we reach a state of herd immunity for future decision-making on national epidemiological control measures.
